# Influencing factors of rehabilitation participation among patients with pneumoconiosis in Chongqing, China

**DOI:** 10.3389/fmed.2025.1575054

**Published:** 2025-07-30

**Authors:** Xiaodong Wang, Lvsu Ye, Lijie Long, Xiaoya Huang, Yongyi Wang

**Affiliations:** ^1^The Affiliated Rehabilitation Hospital of Chongqing Medical University, Chongqing, China; ^2^The First Affiliated Hospital of Chongqing Medical and Pharmaceutical College, Chongqing, China

**Keywords:** pneumoconiosis, influencing factors, rehabilitation, occupational health, public health

## Abstract

**Background:**

Pneumoconiosis is the most serious and common occupational disease in China. This study aimed to analyze the factors influencing participation in pulmonary rehabilitation among patients with pneumoconiosis and provide a basis for decision-making to promote their engagement in rehabilitation.

**Methods:**

We collected data from patients with complete file information between January 2020 and December 2023. The basic information, disease stage, and other characteristics were analyzed. SPSS (version 22.0) statistical software was used for the chi-square test, U-test, and logistic regression analysis.

**Results:**

A total of 1994 patients with pneumoconiosis were included in this study. Of these, 77.8% of the patients participated in rehabilitation and 22.2% did not take part. Comparisons between the two groups revealed that the average annual hospitalization times, hospitalization expenditure, patient income, family expenditure, and disability level in the rehabilitation group were higher than in the non-rehabilitation group (*p* < 0.05). There were statistical differences in household nature, education level, employment status, disease stage, complications, and smoking composition (*p* < 0.05). Logistic regression analysis indicated that patients with urban household registration, smoking history, and complications were less likely to participate in rehabilitation. Conversely, individuals with a higher educational level, advanced disease stage, annual hospitalization expenditure and family expenditure, as well as retired patients were more likely to participate in rehabilitation.

**Conclusion:**

The participation of patients with pneumoconiosis is influenced by various factors, including their household registration nature, educational level, and disease stage. Additional measures should be taken to improve the patients’ motivation for rehabilitation.

## Introduction

Pneumoconiosis represents a major global public health concern. It is a group of occupational lung diseases characterized by diffuse fibrosis of lung tissue resulting from prolonged inhalation and retention of particulate matter in the lungs during occupational activities (1). Studies have shown that the incidence rates of pneumoconiosis are increasing in countries such as the United States, Australia, and others, affecting millions of workers engaged in hazardous occupations around the world (2–4). As a chronic occupational disease, pneumoconiosis has a long incubation period and strong concealment, making recovery difficult and resulting in permanent damage to patients. The associated medical costs, indirect costs, and loss of healthy life years are substantial (5). Since the last century, countries worldwide have increasingly prioritized the prevention and treatment of pneumoconiosis. Countries such as the United States, Australia, Western Europe, and others have implemented strict restrictions or prohibitions on asbestos use. Japan, South Korea, and other countries have formulated laws and regulations related to occupational diseases to strengthen the monitoring of occupational exposure. However, these measures mainly focus on preventing dust-related occupational hazards.

As a nation with a vast population, China has a workforce of >700 million. In 2018, >975,000 cases of occupational diseases were reported in China, with pneumoconiosis accounting for 90% of these cases (6). More than 60% of the global burden of pneumoconiosis occurs in China (7). Therefore, it is of immense economic and social significance to enhance the prevention and treatment efforts for this condition. China has made great efforts in the prevention and treatment of pneumoconiosis, collaborating with institutions, departments, and government levels to assist patients through legislation, regulatory systems, occupational disease prevention and control system, and dust control measures. In July 2019, the National Health Commission of the People’s Republic of China, along with 10 other government departments jointly issued the Action Plan for the Prevention and Treatment of Pneumoconiosis. This plan advocates the establishment of pneumoconiosis rehabilitation stations in key areas, emphasizing rehabilitation treatments for patients. This represents a new exploration of rehabilitation treatment methods for patients with pneumoconiosis.

Chongqing is renowned as an industrial hub in China, including sectors such as chemicals, manufacturing, and IT. Emerging industries and traditional industries coexist, with a complete range of industrial categories. There are 40,890 patients with pneumoconiosis in Chongqing and the direct and indirect economic losses caused by pneumoconiosis are substantial (8). To ensure that patients with pneumoconiosis at the grassroots level receive timely and accessible rehabilitation, Chongqing has taken the initiative to explore the establishment of pneumoconiosis rehabilitation stations in combination with the conditions of township health centers. This initiative has yielded positive outcomes (9). By December 22, 2023, China has successfully established 829 pneumoconiosis rehabilitation stations nationwide, following the Chongqing model. These stations have provided >1.2 million free rehabilitation services to patients, including treadmill, breathing training and so on. Nevertheless, a considerable number of patients still decline participation in rehabilitation treatments and an analysis of the factors influencing their participation in rehabilitation remains missing. Therefore, this study aims to analyze the factors influencing the participation of patients with pneumoconiosis and to provide a decision-making basis for improving the motivation of patients and further enhancing pneumoconiosis rehabilitation efforts.

## Methods

Our data are obtained from the Chongqing pneumoconiosis Rehabilitation Management Information Platform system (From January, 2020 to December, 2023). Patients with complete basic information, including gender, ethnicity, age, education level, smoking history, spouse status, complications, household registration nature, insurance payment category, employment status, disease stage, disability level, average annual number of hospitalizations, average annual hospitalization expenditure, average annual income of patients, and average annual family expenditure, were included in this study. Patients with missing information for any of the aforementioned criteria were excluded from the analysis. A total of 1994 patients were included in this study and all were male participants.

We categorized ethnic groups (Han or ethnic minorities), age groups (≤49, 50–59, 60–69, 70–79, ≥80), education levels (uneducated, primary school, junior high school, senior high school or higher), smoking (yes or no), having spouse (yes or no), complication (yes or no), household registration nature (rural or urban), Insurance payment category (others, medical insurance, employment injury insurance), employment status (unemployed, Retire, employed), staging diagnosis (I, II, III).

The staging diagnosis follows the criteria outlined in the Diagnosis of Occupational Pneumoconiosis (GBZ 70–2015) (10) (Table 1).

**Table 1:** The staging diagnosis of pneumoconiosis.

Staging diagnosis	One of the following criteria must be met
Stage I	Small shadows with an overall density of grade 2, distributed over more than 4 lung regions;
	Exposure to asbestos dust, with small shadows of overall density grade 1, distributed in only one lung region, and pleural plaques simultaneously appearing;
	Exposure to asbestos dust, the overall density of small shadows was 0, but at least two lung regions had a density of 0/1 for small shadows, and pleural plaques appeared simultaneously.
Stage II	Small shadows with an overall density of grade 2, distributed over more than 4 lung regions;
	Small shadows with an overall density of grade 3, distributed 4 lung regions;
	Exposure to asbestos dust, with small shadows of overall density grade 1, distributed over more than 4 lung regions, and pleural plaques that have already affected part of the heart margin or diaphragm surface;
	Exposure to asbestos dust, with small shadows of overall density grade 2, distributed 4 lung regions, and pleural plaques that have already affected part of the heart margin or diaphragm surface.
Stage III	Large shadows appear, with the long diameter not less than 20 mm and the short diameter greater than 10 mm;
	Small shadows with an overall density of grade 3, distributed over more than 4 lung regions and with small shadow clusters;
	Small shadows with an overall density of grade 3, distributed over more than 4 lung regions and with large shadows;
	Exposure to asbestos dust, with small shadows of overall density grade 3, distributed over more than 4 lung regions, and the combined length of a single or multiple pleural plaques on both sides exceeds half of the length of a single chest wall or involves the heart margin, causing partial structural disorganization.

The disability level of pneumoconiosis adheres to the criteria established in Standard for identify work ability-Gradation of disability caused by work-related injuries and occupational diseases (GB/T16180-2014) (11) (Table 2).

**Table 2:** The disability level of pneumoconiosis.

Disability level	Criteria:
Level 1	Stage III pneumoconiosis with severe pulmonary impairment and/or severe hypoxemia [PaO₂ < 5.3 kPa (<40 mmHg)]
Level 2	Stage III pneumoconiosis with moderate pulmonary impairment and/or moderate hypoxemia; Stage III pneumoconiosis with active pulmonary tuberculosis; Stage II pneumoconiosis with severe pulmonary impairment and/or severe hypoxemia [PaO₂ < 5.3 kPa (40 mmHg)]
Level 3	Stage III pneumoconiosis; Stage II pneumoconiosis with moderate pulmonary impairment and/or moderate hypoxemia; Stage II pneumoconiosis with active pulmonary tuberculosis
Level 4	Stage II pneumoconiosis; Stage I pneumoconiosis with moderate pulmonary impairment and/or moderate hypoxemia; Stage I pneumoconiosis with active pulmonary tuberculosis
Level 5	Moderate pulmonary impairment or moderate hypoxemia
Level 6	Stage I pneumoconiosis with mild pulmonary impairment and/or mild hypoxemia
Level 7	Stage I pneumoconiosis with normal pulmonary function

The statistical analysis was conducted using SPSS software (version 22.0). Normal distribution of continuous variables was tested using the Kolmogorov–Smirnov test which indicated non-normal distributions. Therefore, continuous variables were presented as median (minimum-maximum) and analyzed using U-test. Categorical variables were presented as proportions (%) and frequencies (n) and were analyzed using the Chi-square test. Binary logistic regression analysis was used to clarify related influencing factors. All statistical tests were two-sided and *p* values <0.05 were considered statistically significant.

## Results

In this study, a total of 1994 male patients with pneumoconiosis were included, with 1,551 participating in rehabilitation and 443 not participating. The demographic and other characteristics of all patients are summarized in Table 3.

**Table 3:** Demographic and other characteristics of pneumoconiosis patients.

Variables	Total N (%)	Rehabilitation N (%)	Non-rehabilitation N (%)	Statistics	*p* value
Gender (Male)	1,994	1,551 (77.78)	443 (22.22)		
Ethnic group				χ^2^ = 0.846	0.358
Han	1,963 (98.45)	1,529 (98.58)	434 (97.97)		
Ethnic minorities	31 (1.55)	22 (1.42)	9 (2.03)		
Age group				χ^2^ = 1.943	0.746
≤49	122 (6.12)	92 (5.93)	30 (6.77)		
50–59	947 (47.49)	728 (46.94)	219 (49.44)		
60–69	663 (33.25)	526 (33.91)	137 (30.93)		
70–79	229 (11.48)	180 (11.61)	49 (11.06)		
≥80	33 (1.65)	25 (1.61)	8 (1.81)		
Insurance payment category				χ^2^ = 3.346	0.155
Others	6 (0.30)	5 (0.32)	1 (0.23)		
Medical insurance	1,167 (58.53)	891 (57.45)	276 (62.30)		
Employment injury insurance	821 (41.17)	655 (42.23)	166 (37.47)		
Household registration nature				χ^2^ = 4.088	0.043
Rural	1,825 (91.52)	1,430 (92.20)	395 (89.16)		
Urban	169 (8.48)	121 (7.80)	48 (10.84)		
Education level				χ^2^ = 16.645	0.001
Uneducated	176 (8.83)	124 (8.00)	52 (11.74)		
Primary school	1,246 (62.49)	1,000 (64.47)	246 (55.53)		
Junior high school	524 (26.28)	386 (24.89)	138 (31.15)		
Senior high school or higher	48 (2.41)	41 (2.64)	7 (1.58)		
Employment status				χ^2^ = 34.190	<0.001
Unemployed	1,323 (66.35)	998 (64.35)	325 (73.36)		
Retire	452 (22.67)	396 (25.53)	56 (12.64)		
Employed	219 (10.98)	157 (10.12)	62 (14.00)		
Staging diagnosis				χ^2^ = 25.653	<0.001
I	1,082 (54.26)	795 (51.26)	287 (64.79)		
II	662 (33.20)	546 (35.20)	116 (26.19)		
III	250 (12.54)	210 (13.54)	40 (9.03)		
Have spouse				χ^2^ = 0.008	0.930
No	37 (1.86)	29 (1.87)	8 (1.81)		
Yes	1,957 (98.14)	1,522 (98.13)	435 (98.19)		
Smoking				χ^2^ = 43.940	<0.001
No	616 (30.89)	536 (34.56)	80 (18.06)		
Yes	1,378 (69.11)	1,015 (65.44)	363 (81.94)		
Complication				χ^2^ = 104.686	<0.001
No	862 (43.23)	697 (44.94)	165 (37.25)		
Yes	1,132 (56.77)	854 (55.06)	278 (62.75)		
Disability level	4.00 (1.00–7.00)	4.00 (1.00–7.00)	6.00 (1.00–7.00)	Z = −2.336	0.019
Average annual hospital times	1.00 (0.00–17.00)	1.00 (0.00–17.00)	0.00 (0.00–16.00)	Z = −4.776	<0.001
Average annual hospitalization expenditure (10,000 RMB)	0.10 (0.00–10.00)	0.15 (0.00–10.00)	0.00 (0.00–5.00)	Z = −5.107	<0.001
Average annual patient income (10,000 RMB)	2.00 (0.00–13.00)	2.60 (0.00–13.00)	1.60 (0.00–10.00)	Z = −6.833	<0.001
Average annual Family expenditure (10,000 RMB)	3.00 (0.00–30.00)	3.20 (0.00–13.00)	2.30 (0.00–30.00)	Z = −9.370	<0.001

Compared to the non-rehabilitation group, the average annual hospitalization times, average annual hospitalization expenditure, average annual patient income, average annual family expenditure, and disability level in the rehabilitation group were significantly higher (*p* < 0.05). In addition, statistical differences were observed in household registration nature, education level, employment status, disease stage, presence of complications, and smoking status (*p* < 0.05).

Table 4 shows the influencing factors affecting patient participation in rehabilitation. The results of regression analysis suggested that patient engagement in rehabilitation is significantly associated with household registration nature, educational level, employment status, disease stage, smoking status, complications, average annual hospitalization expenditure, and annual family expenditure (*p* < 0.05). Patients with urban household registration, smoking history, and complications were less likely to participate in rehabilitation. Conversely, individuals with higher educational levels, advanced disease stage, higher average annual hospitalization expenditure and family expenditures, as well as retired patients, demonstrated a greater likelihood of participating in rehabilitation (*p* < 0.05).

**Table 4:** Factors influencing participation in rehabilitation among pneumoconiosis patients.

Factors	*β*	Wald	OR (95%CI)	*p* value
Household registration nature				
Rural			1 (reference)	
Urban	−0.584	8.070	0.558 (0.373–0.834)	0.005
Education level				
Uneducated			1 (reference)	
Primary school	0.621	10.314	1.861 (1.274–2.719)	0.001
Junior high school	0.421	3.996	1.523 (1.008–2.300)	0.046
Senior high school or higher	1.092	5.654	2.979 (1.211–7.327)	0.017
Employment status				
Unemployed			1 (reference)	
Retire	0.474	6.624	1.606 (1.120–2.304)	0.010
Employed	−0.210	1.449	0.811 (0.575–1.142)	0.230
Staging diagnosis				
I			1 (reference)	
II	0.464	7.639	1.591 (1.145–2.211)	0.006
III	0.638	6.835	1.893 (1.173–3.054)	0.009
Smoking				
No			1 (reference)	
Yes	−0.551	14.991	0.576 (0.436–0.762)	<0.001
Complication				
No			1 (reference)	
Yes	−0.412	11.891	0.662 (0.524–0.837)	0.001
Disability level	0.073	2.646	1.107 (0.985–1.175)	0.104
Average annual hospital times (times)	0.041	0.998	1.042 (0.962–1.128)	0.318
Average annual hospitalization expenditure (10,000 RMB)	0.266	4.959	1.305 (1.032–1.650)	0.026
Average annual patient income (10,000 RMB)	0.075	2.659	1.078 (0.985–1.181)	0.103
Average annual family expenditure (10,000 RMB)	0.127	12.580	1.135 (1.058–1.218)	<0.001

## Discussion

Due to the prolonged incubation period and the challenging nature of its cure, there will be a substantial number of patients affected with pneumoconiosis in the future. Although the pneumoconiosis rehabilitation stations provide free treatments for patients, there are patients who choose not to participate. Therefore, analyzing the factors influencing patient participation in rehabilitation is crucial for the continuous development of this work. A study demonstrated that diagnostic stage, educational level, and comorbidities or complications were possible influencing factors for the behavior score of pulmonary rehabilitation (12). Our findings indicate that pneumoconiosis patients’ participation in rehabilitation correlates with their household registration status, education level, employment status, disease stage, smoking history, complications, average annual hospitalization costs, and annual household expenditures. Therefore, to enhance the motivation for pneumoconiosis rehabilitation requires the consideration of sociodemographic economic characteristics and the factors related to disease status.

Patients with higher education levels demonstrate greater participation in rehabilitation, which can be explained by the fact that the patients may have a better understanding of the disease and greater motivation to receive health education and rehabilitation treatment. The findings align with Tan’s research (13). Another study of pulmonary rehabilitation participation in COPD patients similarly found that education level influences adherence (14), consistent with our observations. From the perspective of employment status, retired patients are more motivated to participate in rehabilitation. Although the difference lacks statistical significance among working patients, time constraints likely explain this observation. Economically, patients with higher average annual hospitalization expenditure and average annual family expenditure tend to participate more in rehabilitation. This suggests that these patients aim to improve their quality of life through rehabilitation and have a stronger intention to reduce the economic burden on themselves and their families. Previous studies have demonstrated that rehabilitation through pneumoconiosis stations can reduce the number of readmissions and hospitalization expenses for patients with pneumoconiosis (9). Most of the Chinese patients with pneumoconiosis are migrant workers. According to our study, >90% of patients with pneumoconiosis have rural household registrations in Chongqing, and the proportion of these patients participating in rehabilitation is higher than that of urban patients. This may be related to the fact that most pneumoconiosis rehabilitation stations are set up in township health centers in districts and counties. Difficulties with transport and parking costs can affect the progress of rehabilitation (15). Patients living in rural areas have an advantage in terms of distance and economic considerations over those living in urban areas. Due to the convenience of medical treatment, urban patients may be more inclined to go to the nearest hospital for treatment, but patients may incur extra costs for rehabilitation at these locations, thus reducing their chances of participation. Although the number of urban registered patients is relatively small overall, this part of the demographic should not be ignored. Future efforts should focus on strengthening the management of rehabilitation treatments for this population.

Patients’ disease status is an important factor influencing their participation in rehabilitation. Wang et al. (12) observed higher pulmonary rehabilitation behavior score among stage I patients compared to stage II cases, and patients without comorbidities or complications demonstrated lower score than those with comorbidities or complications. Our results indicate that patients at more advanced disease stages demonstrate higher rehabilitation participation rates, while those with complications exhibit lower engagement compared to complication-free patients. This might seem contradictory, as complications typically indicate a more serious condition. At present, the diagnosis of pneumoconiosis in China is mainly based on Diagnosis of Occupational Pneumoconiosis (GBZ 70–2015). Pneumoconiosis is classified into stages I, II, and III, based on the overall density of small shadows, distribution range, presence of large shadows, and pleural spots observed on the chest X-ray film, and the heaviest period is III. This is the grading of imaging. The complications of pneumoconiosis include respiratory system infection, tuberculosis, chronic obstructive pulmonary disease, pneumothorax, chronic pulmonary origin heart disease, and so on (16). This is a clinical diagnosis. Patients with advanced imaging stages may not have complications, and those with complications may not always have more severe clinical manifestations than those without complications. Therefore, it is crucial to not rely on a single factor. In addition, we also considered factors such as disability level and smoking factors. According to the Standard for identify work ability-Gradation of disability caused by work-related injuries and occupational diseases (GB/T16180-2014), pneumoconiosis stage I is associated with a disability level of at least 7. A smaller number indicates a higher disability level, reflecting more severe functional dysfunction. Although the disability grade of patients in the rehabilitation group was higher than that in the rehabilitation group, regression analysis showed no statistical difference. Previous studies have found a positive correlation between silica dust exposure and mortality from respiratory diseases, lung cancer, and cardiovascular diseases (17). Furthermore, there exists an interaction between silica dust exposure and smoking that affects the risk of total and cause-specific mortality (18, 19). Therefore, patients who smoked showed less motivation to recover compared to nonsmokers, and this can be attributed to the effect of smoking on disease severity. In the future, the disease status of patients with pneumoconiosis may need to be further analyzed by combining the disease stage, the severity and category of complications, the disability level, and the smoking factors.

### Limitations of this study

This study had several limitations. Initially, the primary data were obtained from physicians at various pneumoconiosis rehabilitation stations, which may have introduced potential information bias. Additionally, all patients included in this study were male. Although the incidence of pneumoconiosis among females is significantly lower than that of males, the rehabilitation needs of female patients should not be ignored. Factors affecting the participation of patients with pneumoconiosis in rehabilitation, such as disease stage, severity of complications, and smoking, require further detailed analysis and investigation.

## Conclusion

Whether patients with pneumoconiosis participate in rehabilitation is influenced by several factors, including education level, economic situation, and disease stage. Preventing and treating pneumoconiosis is a long-term work requiring the attention and participation of the whole society. In the future, the government should strengthen supervision and investment in the healthcare system, collaborating with social assistance institutions to reduce the economic burden on patients with pneumoconiosis. At the same time, proactive health education efforts should promote lifestyle changes among patients and ensure effective disease treatment and rehabilitation to delay its progression. Finally, a long-term mechanism of government leadership, support by relief institutions, and public participation will be formed, and the work of pneumoconiosis rehabilitation stations should be promoted for a long time, and the rescue and treatment of pneumoconiosis patients should be effectively done. This study provides a decision-making basis for promoting the participation of patients with pneumoconiosis in rehabilitation.

## Data Availability

The raw data supporting the conclusions of this article will be made available by the authors, without undue reservation.

## References

[1] Occupational Lung Diseases Group of Labor Hygiene and Occupational Diseases Branch of Chinese Preventive Medical Association. Consensus of Chinese experts on pneumoconiosis treatment. Environ Occup Med. (2018) 35:677–89. doi: 10.13213/j.cnki.je-om.2018.18437

[2] Blackley DJ Halldin CN Laney AS. Continued increase in prevalence of coal workers’ pneumo-coniosis in the United States, 1970–2017. Am J Public Health. (2018) 108:1220–2. doi: 10.2105/AJPH.2018.304517, PMID: 30024799 PMC6085042

[3] Shi P Xing X Xi S Jing H Yuan J Fu Z. Trends in global, regional and national incidence of pneumoconiosis caused by different aetiologies: an analysis from the global burden of disease study 2017. Occup Environ Med. (2020) 77:407–14. doi: 10.1136/oemed-2019-106321, PMID: 32188634

[4] Zosky GR Hoy RF Silverstone EJ Brims FJ Miles S Johnson AR. Coal workers' pneumoco-niosis: an Australian perspective. Med J Aust. (2016) 204:414–8. doi: 10.5694/mja16.00357, PMID: 27318401

[5] GBD 2016 Occupational Chronic Respiratory Risk Factors Collaborators. Global and regional burd-en of chronic respiratory disease in 2016 arising from non-infectious airborne occupational exposur-es: a systematic analysis for the global burden of disease study 2016. Occup Environ Med. (2020) 77:142–50. doi: 10.1136/oemed-2019-106013, PMID: 32054818 PMC7035690

[6] Lancet T. Improving occupational health in China. Lancet. (2019) 394:443. doi: 10.1016/s0140-6736(19)31799-4 31402011

[7] Li J Yin P Wang H Wang L You J Liu J. The burden of pneumoconiosis in China: an analysis from the global burden of disease study 2019. BMC Public Health. (2022) 22:1114–24. doi: 10.1186/s12889-022-13541-x, PMID: 35659279 PMC9166455

[8] Luo D Liu YS Wang W Wang YY Wang SS. Practice and exploration of the pilot construction of pneumoconiosis rehabilitation station in Chongqing township health center. Chinese J Indu-strial Med. (2022) 35:94–5. doi: 10.13631/j.cnki.zggyyx.2022.01.035

[9] Bai L Liu YS Luo D. Chongqing pneumoconiosis rehabilitation station study on reducing direct economic loss of patients. Occup Health & Emerg Rescue. (2023) 41:137–139+171. doi: 10.16369/j.oher.issn.1007-1326.2023.02.003

[10] National Health and Family Planning Commission of the People's Republic of China. Diagnosis of occupational pneumoconiosis. Beijing, China: Standards Press (2015).

[11] Ministry of Human Resources and Social Security of the People's Republic of China. Standard for identify work ability—Gradation of disability caused by work-related injuries and occupational diseases. Beijing, China: Standards Press (2014).

[12] Wang JY Liu J Xie Y. Investigation on KAP status and influencing factors of pulmonary reha-bilitation among pneumoconiosis patients in Shenzhen. Occup Health & Emerg Rescue. (2024) 42:580–6. doi: 10.16369/j.oher.issn.1007-1326.2024.05.004

[13] Tan LH. Exploration of health education content for inpatients with coal workers' pneumoconiosis. Chin J Coal Ind Med. (2014) 17:760–3. doi: 10.11723/mtgyyx1007-9564.2014.05.027

[14] Xia X Xia K Yao X Song J Liu Y Liu X. Factors influencing compliance with pulmonary rehabilitation in patients with stable COPD: a cross sectional study. Int J Chron Obstruct Pulmon Dis. (2025) 20:895–904. doi: 10.2147/COPD.S506248, PMID: 40191264 PMC11970269

[15] Hug S Cavalheri V Gucciardi DF Hill K. An evaluation of factors that influence referral to pulmonary rehabilitation programs among people with COPD. Chest. (2022) 162:82–91. doi: 10.1016/j.chest.2022.01.006, PMID: 35032478

[16] Wang H Lyu X Luo D Dai H. Prevalence and types of comorbidities in pneumoconiosis China, 2018-2021. China CDC Wkly. (2023) 5:837–43. doi: 10.46234/ccdcw2023.159, PMID: 37814646 PMC10560375

[17] Chen W Liu Y Wang H Hnizdo E Sun Y Su L. Long-term exposure to silica dust and risk of total and cause-specific mortality in Chinese workers: a cohort study. PLoS Med. (2012) 9:e1001206. doi: 10.1371/journal.pmed.1001206, PMID: 22529751 PMC3328438

[18] Lai H Liu Y Zhou M Shi T Zhou Y Weng S. Combined effect of silica dust exposure and cigarette smoking on total and cause-specific mortality in iron miners: a cohort study. Environ Health. (2018) 17:46–56. doi: 10.1186/s12940-018-0391-0, PMID: 29743082 PMC5943994

[19] Wang D Yang M Liu Y Ma J Shi T Chen W. Association of Silica Dust Exposure and Cigarette Smoking with Mortality among Mine and Pottery Workers in China. JAMA Netw Open. (2020) 3:e202787. doi: 10.1001/jamanetworkopen.2020.2787, PMID: 32286660 PMC7156992

